# STORIES statement: Publication standards for healthcare education evidence synthesis

**DOI:** 10.1186/s12916-014-0143-0

**Published:** 2014-09-03

**Authors:** Morris Gordon, Trevor Gibbs

**Affiliations:** School of Medicine and Dentistry, University of Central Lancashire, Fylde road, Preston, PR1 2HE UK; Department of Child Health, Blackpool Victoria Hospital, Blackpool, UK; National Medical Academy of Postgraduate Education, Kiev, Ukraine; Association of Medical Education in Europe, Dundee, UK

**Keywords:** Evidence synthesis, Systematic review, Secondary research, Evidence based medicine, Evidence based education

## Abstract

**Background:**

Evidence synthesis techniques in healthcare education have been enhanced through the activities of experts in the field and the Best Evidence Medical Education (BEME) collaborative. Despite this, significant heterogeneity in techniques and reporting of healthcare education systematic review still exist and limit the usefulness of such reports. The aim of this project was to produce the STORIES (STructured apprOach to the Reporting In healthcare education of Evidence Synthesis) statement to offer a guide for reporting evidence synthesis in health education for use by authors and journal editors.

**Methods:**

A review of existing published evidence synthesis consensus statements was undertaken. A modified Delphi process was used. In stage one, expert participants were asked to state whether common existing items identified were relevant, to suggest relevant texts and specify any items they feel should be included. The results were analysed and a second stage commenced where all synthesised items were presented and participants asked to state whether they should be included or amend as needed. After further analysis, the full statement was sent for final review and comment.

**Results:**

Nineteen experts participated in the panel from 35 invitations. Thirteen text sources were proposed, six existing items amended and twelve new items synthesised. After stage two, 25 amended consensus items were proposed for inclusion. The final statement contains several items unique to this context, including description of relevant conceptual frameworks or theoretical constructs, description of qualitative methodologies with rationale for their choice and presenting the implications for educators in practice of the results obtained.

**Conclusions:**

An international expert panel has agreed upon a consensus statement of 25 items for the reporting of evidence synthesis within healthcare education. This unique set of items is focused on context, rather than a specific methodology. This statement can be used for those writing for publication and reviewing such manuscripts to ensure reporting supports and best informs the wider healthcare education community.

**Electronic supplementary material:**

The online version of this article (doi:10.1186/s12916-014-0143-0) contains supplementary material, which is available to authorized users.

## Background

Evidence-based health care involves the systematic collection, synthesis and application of all available scientific evidence, not just the opinion of experts [1]. The integration of this concept into health care over the last 30 years represented a shift from a position of expert based consensus guidance to evidence led guidance for evolving clinical knowledge [2]. The most important element of the evidence-based health care movement is an acceptance of the evolving nature of clinical truth. Researchers have sought to quantify this, none more elegantly than Hall and Platell [3]. They demonstrated that the half-life of clinical truth in the surgical field is 45 years and, therefore, within half a century 50% of what is known is wrong. This more than anything cements the need for a contemporaneous and evidence-based knowledge base [4].

For more than a decade, there have been calls for medical education to become more evidence-based [5-7]. In particular, the question of quality has been a key focus [8], with concern raised regarding a lack of methodological rigour compared to the accepted hierarchies of evidence in clinical medicine research. Responses from the field highlighted that medical education research ‘cannot be viewed in such a uni-dimensional way’ [9] and essentially suggests that evidence should not be viewed in hierarchies of quality but should be selected like colours in a rich tapestry [10]. Paradoxically, this means that in the field of medical education, the issues of evidence synthesis are far more complex and challenging.

The Best Evidence Medical Education (BEME) collaborative was established in 1999 [11], rejecting the use of anecdotal evidence in medical education and concentrating on the use of evidence synthesis through systematic review. The focus of early activity was to establish a process to undertake such evidence synthesis that is inclusive of the unique study aims, designs and outcome measures of medical education research. It is not surprising that early BEME reviews identified the same issues of poor quality within primary educational research that had been recognised within the wider medical education literature [8].

Since the inception of BEME, the conversations within the medical education field have radically changed, with a new zeitgeist disseminating through such research. Key to this revolution has been substituting the often self evident question as to ‘whether’ education interventions are effective [12] and focusing on educational research outcomes that are likely to influence teaching practice [13], such as studies asking ‘how’, ‘why’, ‘when’ and ‘for whom’ [14]. This shift from ‘justification’ studies to ‘descriptive and clarification’ studies [15] delivers a clearer picture to allow replication of teaching practice and a deeper theoretical understanding of the issues at play to guide future educational innovations [16].

The challenge for those completing secondary evidence synthesis within healthcare education has been to evolve techniques that can reflect this revolution in primary education research. Organisations such as BEME have embraced such changes, leading to rapidly updated author guidance, as well as current structural reorganisations to widen methodological and general education expertise within the organisation [17]. Scholars in the field are openly discussing methodology that can be employed to synthesise such evidence, integrating qualitative synthesis techniques into medical education systematic review [18]. However, the situation within the wider literature is starkly conflicting, with education systematic review manuscripts often still entrenched in questions of ‘whether’ education is effective, offering little to readers, educators and policy makers [19].

An example can be found in the often cited landmark systematic review on Internet based medical education published in 2008 which concluded that e-learning is better than no intervention and similar (on average) to traditional instruction [20]. Whilst this does indeed reflect the focus of much primary research and so is an accurate statement, this clearly is not a useful conclusion, once characterised as like comparing printing press-based and quill-based forms of learning [21]. Specific opportunities missed within this review were to highlight that the actual ‘learning’ is most often absent in many included studies, with little or no description of the instructional objectives, pedagogical basis, resources required or methods of design [22]. The authors published a far more illuminating follow up piece describing the constituents of the education described in the primary research [23].

Publication standards are common, useful and often required by those conducting health research. Examples include CONSORT for randomized controlled trials [24], PRISMA for Cochrane-style systematic reviews [25], SQUIRE for quality improvement studies [26] and RAMESES for realist reviews [27]. In the context of healthcare education systematic review, with the complexities of primary studies and the difficulties and choices of methodology highlighted, such a statement is particularly needed. This would support authors in reporting and planning work that is likely to offer the most to readers. Such a statement would also support peer reviewers and journal editors who are often working in subject specific fields and, as such, may not have full insight into what constitutes high quality educational systematic review. Finally, such a statement will support readers by offering a clear and concise set of criteria with which they can consider the quality of a piece of healthcare education evidence synthesis.

This study set out to develop the STORIES (STructured apprOach to the **R**eporting **I**n healthcare education of Evidence Synthesis) statement as a baseline set of reporting criteria.

## Methods

This study was approached in three phases. Firstly, a review of existing evidence publication standards, statements or checklists for evidence synthesis and systematic review, with key items extracted to make a set of consensus core items for inclusion. Secondly, a three-round Delphi method with an interdisciplinary panel of national and international experts in medical education evidence synthesis, policy and/or publishing was used to produce and refine a set of methodological items and publishing standards. Finally, the draft STORIES statement was used to assess contemporaneous medical education systematic reviews, both to assess whether it is fit for purpose or to guide refinements as needed. Prior to commencement, the protocol was presented to the BEME editorial board for informal peer review.

### Review of existing standards

A literature review using the following search strategy: (‘systematic review’ OR ‘meta-analysis’ OR ‘evidence synthesis’ OR ‘publication standards’) AND (‘checklist’ OR ‘reporting’ OR ‘statement’) was undertaken in the Medline database from 1993 to the present day. Papers reporting a standardised set of criteria for any form of evidence synthesis in healthcare were included. Four such publications were deemed relevant [25,27-29]. A fifth paper was excluded as it reported the QUOROM statement [30], which was a precursor from the group who went on to develop the included PRISMA statement [25] and they are essentially homogenous. Analysis of these checklists found a key list of consistent items occurring in all such statements, presented below.

Common items to reported systematic review statements/checklists

• Describe as a systematic review piece, with specific type mentioned

• Provide a structured summary

• Describe the rationale for the review in the context of what is already known

• Provide an explicit statement of questions being addressed

• State why this method of review was selected

• State and provide a rationale for how the searching was done

• Provide details on all the sources of information and dates searched

• Electronic database details should include full search terms for at least one database

• If individuals familiar with the relevant literature and/or topic area were contacted, indicate how they were identified, selected, contacted and what they contributed

• Explain how judgements were made about inclusion/exclusion

• Describe the process of data extraction and any process of contacting authors for confirmation of/or more data

• Describe and justify the method of analysis and how quality was assessed

• Give a flow diagram summarising study selection

• Provide the characteristics of all included documents

• Present the main findings in light of the reviews objectives

• Discuss strengths and limitations of the review and its findings, commenting on the strength of the evidence

• Give guidance for future research

• Provide details of funding

These items were presented as a bare minimum set of reporting items for any evidence synthesis.

### Delphi process

An online Delphi method was used. Three authors of prior published BEME systematic reviews volunteered to pilot the survey, with amendments and suggestions incorporated. Members of the expert panel were then recruited through several methods. Firstly, two additional members of the BEME editorial board were recruited. Secondly, invitations were sent to all corresponding authors for published BEME reviews. Finally, all corresponding authors for evidence synthesis publications within key journals were invited to take part. Articles from August 2012 to July 2013 were considered through hand searching in the following journals: *Medical Education*, *Medical Teacher*, *Postgraduate Medical Journal*, *Academic Medicine*, *Advances in Health Sciences Education: Theory and Practice*, *BMC Medical Education*, *British Medical Journal*, *Journal of the American Medical Association*, *Lancet* and *New England Journal of Medicine*.

The Delphi panel was conducted online using the online survey tool, ‘surveymonkey’. It began with a ‘brainstorm’ round (‘round 1’). Participants were sent the preliminary consensus item list and asked to comment on the suitability of these items for inclusion. Additionally, expert participants were invited to submit personal views, exchange theoretical and empirical papers on the topic and suggest items for inclusion. These were collated by the authors in preparation for the next round (‘round 2’). The final list of consensus items was presented, as well as new amended items, with participants asked ‘yes/no’ for inclusion. Each of the suggested resources, as well as empirical items for inclusion, were presented as a questionnaire with likert scales to indicate relative importance validity and sent to participants for ranking (‘round 2’). Once again, the responses were collated by the authors and as consensus was reached on the majority of items, a final first draft of the statement was sent to participants for review.

### Piloting and refinement

The statement was used to assess a contemporaneous published medical education systematic review from a leading journal by local colleagues and collaborators representing various professions, including nursing, medicine, health informatics and allied health professionals. Additionally, panel members were invited to review the same study and consistency of responses analysed, as well as free text feedback on the statement collated.

## Results

A total of 35 potential expert panel members were identified to invite for participation – 10 authors of BEME reviews and a further 25 from published reviews within the hand searched journals. Within this group were two members of the BEME editorial board and so no further BEME members were invited to participate. Nineteen experts agreed to take part and completed the Delphi survey (54%), with four continents represented (seven – UK, four – Australia, three – Canada, two – USA, one – Ukraine, one – Pakistan, one – Netherlands). The Delphi panels ran between September 2013 and January 2014. All participants completed all the questions in round 1. This was also the case in round 2, with 100% response in all sections of the survey. Feedback leading to changes of the final document was received from four experts, with no further concerns raised from the other experts. The statement was sent out to the panel for piloting with a contemporaneous published education systematic review. This led to a 75% overall agreement in judgements made by returning panel members. Additionally, there was universal feedback that the STORIES statement supported the judging of the report.

The STORIES statement is presented in Additional file 1 and is available for download directly [31].

### How to use the STORIES statement

The structure of the STORIES statement has drawn on previous methodological publications and, in particular, on the ‘Explanations and Elaborations’ document of the PRISMA statement [25] and recently reported RAMESES standards [27]. Each item is followed by an explanation, which gives insights into the panel discussions and rationale, as well as, often, an example drawn from published reviews. The standards set out what might be expected for reporting, but given the massive range of methodologies and primary evidence available for such reviews, authors and peer reviewers will still need to take a view on the level of detail and pertinence of each item. The order in which items are reported may vary. Realist syntheses are not ‘linear’ reviews.

#### Title

**1. Use a title that includes a description of the aims of the piece (educational effectiveness, descriptive, and so on) and the method of evidence synthesis (for example, realist, meta-ethnographic, and so on.)**

##### Example

‘Internet-based medical education: a realist review of what works, for whom and in what circumstances.’ [32].

##### Explanation

In is clear that a title for evidence synthesis has multiple purposes in this context. It was apparent from the first part of this study that within healthcare education this is still often not clearly indicated by authors and only implicit. The primary requirement is to set out the aims of the study and as such tell readers whether the focus of the review is one of description, justification or clarification [15]. Additionally, our expert panel also flagged up the importance of also reflecting the specific review methodology, as there is such a wide range on offer within the field [18].

#### Abstract

**2. Provide a structured summary**

##### Explanation

This item was consistent across all other publication standards for evidence synthesis. In the context of education evidence synthesis, this should seek to highlight the key items from the statement. In particular, the implications of the work for future educational research (item 22) and the implication for educators (item 23).

#### Introduction

**3. Describe the rationale for the review in the context of what is already known**

**4. Provide a statement of the questions being addressed by the study**

##### Example

‘There has not been a systematic review of the literature on teaching professionalism. The heterogeneity of learning theories and teaching approaches employed makes such a review a difficult undertaking. This difficulty is compounded by the varying ways that professionalism has been defined and the lack of consensus on what criteria make up medical professionalism.’

‘Our research question was: What teaching processes, systems, and approaches have been found to work to ensure an ethos of professionalism in medical graduates? We sought to discover: What works in teaching professionalism? (Method), How does it work? (Methodology), Why does it work? (Theory) and What does it teach?*’* [33].

##### Explanation

Given the massive and often heterogeneous evidence base that exists within healthcare education research, it is key for the study to address both why the review is being completed (by discussing what is the current state of the field) and by addressing the context for this particular review. This may include justification as to why specific learner groups, educational environments or content topics are being selected for this piece of work and how this relates to existing works, possibly within other fields.

The expert panel also expressed a strong desire for a specific set of questions to be stated as the aims for the work and for there to be a match between these questions, the title of the review (item 1) and the methods of synthesis (item 5). Whilst this may seem implicit, it is clear that often these three elements are not aligned [19,34]. This may reflect an evolution in aims of the study that has been led by emerging data, but it is important that a retrospective report explains this in a transparent manner (item 21). Additionally, given the wide range of aims of review within healthcare education, it is important to clearly signpost for the reader what this study will tell them. Will the review describe education or curricula items, address effectiveness or look to clarify understanding?

**5. State why this method of evidence synthesis was selected within the context of the questions being asked**

##### Example

‘A realist review using Pawson’s techniques of realist enquiry to include a wider range of outcomes and criteria for inclusion including grey literature and other non-empirical reports and documents. This would consider the complexity and other phenomena associated with CEME. A realist synthesis review attempts to answer the question “how and when does it work and for whom”, in doing so it draws on a wider range of sources than a systematic review and it is based on synthesizing commentaries rather than ratings. Again we would seek to answer the following core questions: What key factors define CEME? What are the strengths and limitations of CEME?’ [35].

##### Explanation

In keeping with items 3 and 4, as well as stating a clear objective and why this has been selected, authors must particularly relate these to the methods of evidence synthesis selected. This is not because such decisions can be judged as right or wrong, as it is entirely reasonable to approach the same review questions with different methodologies. Given this diverse range of methodologies and the various epistemological stances that can be taken by authors, understanding the decisions that have been made allows readers to more fully interpret the results and conclusions of the study [10].

#### Methods

**6. State and provide a rationale for how the searching was done**

**7. Provide details on all the sources of information and dates searched**

**8. Electronic databases - provide full search terms for at least one database, with details of deviations in subsequent searches**

##### Example

‘Our objectives for evidence synthesis were not aligned with a particular epistemological stance and thus we did not take a strict positivist or constructionist approach. Rather, we followed a pluralistic model, not using a single arbiter for quality assessment and including a mixture of evidence types.

The following online databases were searched to June 2011 using a standardised search strategy (Appendix online).’ [36].

##### Explanation

As with many of the areas discussed, our panel did not feel that there were any specific limitations or expectations for searching that should be imposed, merely a description should be offered with the rationale for such decisions to support the readers in understanding the breadth of data that the search is likely to cover. As with all evidence synthesis, sufficient detail should be given to support replication.

**9. Describe the process of data extraction and any process of contacting authors for confirmation of/or more data**

**10. Explain the method for judging inclusion/exclusion**

##### Example

‘If a study reported an intervention in limited detail or commented on improved handover without presenting evidence in support of the improvement, we attempted to contact the author for further details’ [37].

##### Explanation

Common with evidence synthesis in other contexts, if methodological data is missing so as to limit judgments on quality, then attempts should be made to contact the authors. However, it is also commonplace in educational systematic review to find that published educational interventions lack the detail to understand fully the education in question. This may consist of a lack of detail as to the structure, resources or learning outcomes and can limit usefulness for readers [34] when synthesizing as part of a descriptive review. This is well recognized in all fields reporting non-pharmacological interventional research, but it is also recognised that in the majority of situations, authors will provide such materials if asked [38]. It may be considered too high a standard to expect such contacts to occur, but if no such attempts are made, authors should be clear as to how they will report such weaknesses in the primary data.

Similarly to other methodological areas, the panel did not feel prescriptive guidance on inclusion and exclusion of studies based on any set criteria was needed, but simply for authors to clearly highlight any decisions or assumptions made to readers.

**11. If quality appraisal tools are used, please describe and justify their choice**

##### Explanation

These items were the focus of much discussion amongst the panel and highlight some of the issues in reporting that led to the conception of these works. There are many different appraisal tools that have been applied in this context [11,39-41]. Authors should describe any such tools that are directly applied or inform such quality judgments. In particular, if decisions on inclusion of studies for synthesis are related to quality judgements, the rationale for the threshold for inclusion must be presented.

**12. Describe qualitative methods for synthesising primary evidence (where appropriate) and the goal of these methods, such as thematic analysis; meta-ethnography, and realist synthesis**

##### Explanation

Qualitative synthesis combines rigorous processes and authorial judgement to present the collective meaning of research outputs [18]. As with primary educational research, those completing evidence synthesis may choose to approach qualitative analysis using any number of methodologies. It is key for descriptions of such methods to be reported by authors with the same rigor as with quantitative methodologies. Several members of the panel reflected that such methodological choice should be related back to the goals of the work and in doing so, justify the choices made.

**13. Describe quantitative methods for synthesising primary evidence (where appropriate), such as meta-analysis and how issues of heterogeneity will be considered**

##### Example

‘We planned for both quantitative and qualitative evidence synthesis. Because there was high between-study inconsistency in prior analyses, we planned to use random effects meta-analysis to quantitatively pool results, organized by comparison (comparison with no intervention, another form of technology-enhanced simulation, or another form of instruction). Additionally, we planned subgroup analyses based on trainee level, topic, and the key instructional design features noted above. Finally, we conducted subgroup analyses based on key study design elements (randomization and blinding of assessment) and sensitivity analyses, excluding the results of studies with imprecise effect size calculations.’ [42].

##### Explanation

The role of quantitative methods of synthesis, such as meta-analysis, within the context of education systematic review is difficult to define. In many cases, the naturally heterogeneous nature of educational interventions and learner groups limits the scope for such analysis. However, when reporting such work, it is important for authors to report in what circumstances they would have used such methods and if indeed they were used, what measures were taken to address heterogeneity. The example above eloquently describes methods that consider the three main forms of heterogeneity that should be accounted for: educational, methodological and statistical heterogeneity.

#### Results

**14. Give a flow diagram summarising study selection**

**15. If individuals familiar with the relevant literature and/or topic area were contacted, provide a summary of the contact and information obtained**

**16. Provide summarised details of included works, considering elements such as methodology, key results and conclusions**

**17. Describe methods of quality assessment of education reported, including all parameters considered (for example, details of study theoretical underpinning, pedagogical strategies and details of teaching activities to allow replication or dissemination)**

**18. Describe quality assessment of the research methods of included studies**

**19. Present the results of qualitative and/or quantitative evidence synthesis**

##### Explanation

all reported evidence synthesis guidance and was agreed by the panel unanimously for inclusion. Item 15 reports the outcomes of item 9 and, as such, was agreed for inclusion by the panel.

The focus of panel discussions was to what extent included works should be presented. It was felt that three broad areas should be reported. These are a summary of the educational research activity itself (item 16), a summary of the education employed in studies (item 17) and a summary of the quality of research methodology of primary studies (item 18). Consistent with other items, the panel avoided prescriptive items and, therefore, attempted to provide broad guidance (Items 16 and 18).

Item 17 gives examples of potential areas to report when discussing actual educational interventions from primary studies. Whilst it could be argued that if a review is focused on effectiveness (justification), such information is not required, panel members argued that having a piece of evidence synthesis that is educational in nature and not presenting any information to inform the reader as to what such education looks like is a missed opportunity [19]. Additionally, it is well recognised that such studies are often devoid of such reporting and highlighting this to the reader [34,38].

#### Discussion

**20. Present the main findings in light of the review objectives**

##### Explanation

Whilst discussing the findings of the review it is key to relate this to the review objectives. This will often be a superfluous item for authors, but the panel noted that it is possible in the context of certain review methodologies, such as realist reviews [27], that the focus of the objectives may have changed in response to the emerging data. In such cases, highlighting how the data informed such changes and what deviations to the protocol must be reported.

**21. Discuss strengths and limitations of the review and its findings, commenting on the strength of the evidence**

##### Example

‘Overall, inconsistencies were evident in methodological reporting and quality, in the 14 studies included in this review. None of the studies reviewed provided an appropriate framework for defining, measuring or understanding emotional intelligence (EI) within their work. This resulted in the inclusion in this review of a wide range of EI proxy measures, thus illustrates the problems caused by the broad definition of EI and related constructs within medical education.’ [43].

##### Explanation

Commenting on the limitations of the review was common to all other published guidance. The panel highlighted that such discussions should particularly relate how the quality of primary extracted data has impacted, and possibly limited, the strength of conclusions made.

**22. Discuss how the findings of the evidence synthesis impact future primary research**

##### Example

‘We have several recommendations for improving the research base for journal clubs. The study design should be based on pedagogical theory, for example using the principles of adult learning.

Each element of the intervention needs to be operationalised with sufficient definition of the variables to facilitate comparison of the active ingredients within the delivery framework. Evaluation questions need to be matched to the goals of the intervention, to the effectiveness measures, and to the level of effect.’ [44].

##### Explanation

This was a common item from existing guidance, but the panel reflected that they thought it was in many ways more important in the context and generally poorly reported by authors. The review team have an extremely in depth knowledge of the state of the field and, as such, are very well placed to highlight explicitly directions for future work. This is one of the least objective and paradoxically most important elements of the reported work.

**23. Describe possible implications of the findings for educators**

##### Explanation

The panel once again reflected that whilst a rather self evident item, this is often missing from existing reviews. Insights should not be limited to the clinical teacher, but where appropriate, give suggestions for curriculum developers and educational policy makers. This allows reviews to be relevant at both the micro and macro educational level.

#### Other

**24. Provide details of funding**

**25. Describe the skills and expertise of the review team and acknowledge any outside help**

##### Explanations

These final items were the most contentious amongst the panel. Some felt them to be unnecessary, but in the final stages of the process, the case for inclusion was decided to be strong. Item 24 was argued to be crucial in all scholarly endeavours. Whilst education is often felt to be less at risk from conflicts of interest and the influence of commercial interests, the nature of granted educational work could still present a potential source of bias and, as such, should be transparently reported to all. Finally, item 25 is a requirement when completing works for organisations such as BEME and Cochrane. The panel did not feel this was primarily to judge whether a team should have completed a review, but to allow readers to frame methodological choices within the context of the team’s expertise.

## Discussion

The STORIES statement has been produced through a multistage process that involved synthesis of existing consensus guidance, a Delphi process and finally the piloting of the draft statement. The aim of this process has been to support reporting of healthcare evidence synthesis that is more informative to researchers, educators and policy makers.

The STORIES statement has clearly been built on previous consensus statements and, as such, it is interesting to consider it within this context (Additional file 2). Whilst a number of the items from previous statements have been retained, a third of the items are either significantly reworked for the health education setting or completely novel (Additional file 2). These items particularly support the collection of data unique to education (items 12 and 17) and ensure the utility of this data is made clear (Items 19 and 23).

Additionally, the retooling of some items to the education context may have a significant impact on the utility of evidence synthesis. For example, the changes in item 1 (title) and item 11 (choosing from a range of quality appraisal tools) both reflect the profoundly multidisciplinary nature of health education and the range of methodologies employed by researchers in the field. In this manner, the STORIES statement is completely unique, focusing on the context of the synthesis, rather than methodology.

The STORIES statement cannot replace primary methodological guidance on how to carry out health education evidence synthesis, but through the expert Delphi panel, a number of key relevant resources have been highlighted for authors. Given the vast array of evidence available in health education research, the kaleidoscopic nature of the evidence and the significant range of valid methodological choices facing authors, the STORIES statement is presented as a framework to support clear reporting. STORIES is not meant to be a prescriptive checklist, but a tool to support authors in considering the choices they have made and to act as a prompt to support transparency in reporting these choices. Many items simply highlight the existence of choice and so it is left to the individual acumen of authors, reviewers and readers to make and judge specific decisions made.

In formulating the STORIES statement, an expert panel was selected that represented a range of professionals working in a vast array of educational settings. The common skill of all panel members was practical experience completing evidence synthesis. As such, it is hoped that the finished statement is pragmatic, grounded in the realities facing authors and reflecting needs with the field.

The STORIES statement is not based on any new empirical works. The outcome of the process is nothing but opinion and the results of the process are only as valid as the opinions of the experts who formed the panel. Whilst the panel did represent those publishing within the peer reviewed literature, there are notable gaps in the range of expertise represented. For example, there is a predominance of medicine educators and a lack any PHD medical educators. This may not invalidate the statement, but limit our ability to comment in any meaningful way on its application within other contexts.

The Delphi process employed a bare consensus set of reporting items within stage one. It can be considered a limitation that this new set of reporting criteria is grounded within previous consensus statements that had ultimately been judged as incomplete within the medical education setting. However, given that the majority of such items were accepted by our panel, it is our assertion that these items are indeed a pragmatic and appropriate backbone for evidence synthesis reporting. Indeed, the value of the STORIES statement is in clarifying that this is the case within the medical education context and the addition of several novel high value items that are situation specific. The STORIES statement is presented not as a final product, but in response to a need. Future work is needed from researchers and clinical teachers to comment on, refine or possibly reject STORIES.

## Conclusions

This study has sought to gain a consensus for reporting of health education evidence synthesis. A review of existing guidance, followed by the convening of a Delphi process involving an international expert panel, has agreed upon a consensus statement of 25 items for reporting. This unique set of items is focused on context, rather than a specific methodology and as such can be applied to all evidence synthesis works within health education. This statement can be used for those writing for publication and reviewing such manuscripts to ensure that reporting supports and best informs the wider healthcare education community.

## Additional files

Additional file 1
**The STORIES statement.**
Additional file 2
**Comparison of STORIES statement with existing evidence synthesis checklists.**

## Abbreviations

BEME: best evidence medical education
CONSORT: Consolidated Standards of Reporting Trials
MOOSE: Meta-analysis Of Observational Studies in Epidemiology
PRISMA: Preferred Reporting Items for Systematic Reviews and Meta-Analyses
RAMESES: Realist And MEta-narrative Evidence Syntheses: Evolving Standards
STORIES: structured approach to the reporting in education of evidence synthesis
