# Supplementary material for: STORIES statement: Publication standards for healthcare education evidence synthesis
Source: BMC Med. 2014 Sep 3;12:143. doi: 10.1186/s12916-014-0143-0 (PMC4243720; doi:10.1186/s12916-014-0143-0)
Supplement: Additional file 1 — The STORIES statement. [file 12916_2014_143_MOESM1_ESM.doc]

| **Checklist item** | | **Page/section and comments** |
| --- | --- | --- |
| **Title** | |  |
| 1 | Use a title that includes a description of the aims of the piece (educational effectiveness, descriptive, etc) and method of evidence synthesis (e.g. realist, meta-ethnographic, etc) |  |
| **Abstract** | |  |
| 2 | Provide a structured summary |  |
| **Introduction** | |  |
| 3 | Describe the rationale for the review in the context of what is already known |  |
| 4 | Provide a statement of the questions being addressed by the study |  |
| 5 | State why this method of evidence synthesis was selected within the context of the questions being asked |  |
| **Methods** | |  |
| 6 | State and provide a rationale for how the searching was done |  |
| 7 | Provide details on all the sources of information and dates searched |  |
| 8 | Electronic databases - provide full search terms for at least one database, with details of deviations in subsequent searches |  |
| 9 | Describe the process of data extraction and any process of contacting authors for confirmation of / or more data |  |
| 10 | Explain the method for judging inclusion / exclusion |  |
| 11 | If quality appraisal tools are used, please describe and justify their choice |  |
| 12 | Describe qualitative methods for synthesising primary evidence (where appropriate) and the goal of these methods, such as thematic analysis; meta-ethnography, and realist synthesis |  |
| 13 | Describe quantitative methods for synthesising primary evidence (where appropriate), such as meta-analysis and how issues of heterogeneity will be considered |  |
| **Results** | |  |
| 14 | Give a flow diagram summarising study selection |  |
| 15 | If individuals familiar with the relevant literature and/or topic area were contacted, provide a summary of the contact and information obtained |  |
| 16 | Provide summarised details of included works, considering elements such as methodology, key results and conclusions |  |
| 17 | Describe methods of quality assessment of education reported, including all parameters considered (e.g. Details of study theoretical underpinning, pedagogical strategies and details of teaching activities to allow replication or dissemination) |  |
| 18 | Describe quality assessment of the research methods of included studies |  |
| 19 | Present the results of qualitative and/or quantitative evidence synthesis |  |
| **Discussion** | |  |
| 20 | Present the main findings in light of the review objectives |  |
| 21 | Discuss strengths and limitations of the review and its findings, commenting on the strength of the evidence |  |
| 22 | Discuss how the findings of the evidence synthesis impact future primary research |  |
| 23 | Describe possible implications of the findings for educators |  |
| **Other** | |  |
| 24 | Provide details of funding |  |
| 25 | Describe the skills and expertise of the review team and acknowledge any outside help |  |

**Key reference works**

- Bearman, M. and Dawson, P. Qualitative synthesis and systematic review in health professions education. Medical Education, 2013; 47: 252–260.
- Greenhalgh T, Peacock R. Effectiveness and efficiency of search methods in systematic reviews of complex evidence: audit of primary sources. BMJ 2005;331:1064–5
- Hammick M, Dornan T, Steinert Y. Conducting a best evidence systematic review. Part 1: From idea to data coding. BEME Guide No. 13. Medical Teacher, 2010; 32:3-15

**Other supporting reference works**

- Boland A, Cherry MG, Dickson R. Doing a systematic review: a student's guide. Sage, 2013.
- Booth A, Papioannou D ,Sutton A. Systematic Approaches to a Successful Literature Review. Sage, 2012.
- Brown PA, Harniss MK, Schomer KG, Feinberg M, Cullen NK, Johnson KL. Conducting systematic evidence reviews: core concepts and lessons learned. Arch Phys Med Rehabil 2012; 93:S177-84.
- Cook DA, West CP. Conducting systematic reviews in medical education: a stepwise approach. Med Educ. 2012 Oct;46(10):943-52
- Cook DA. Narrowing the focus and broadening horizons: complementary roles for systematic and nonsystematic reviews. Adv Health Sci Educ Theory Pract. 2008 Nov;13(4):391-5.
- Crowther MA, Cook DJ. Trials and Tribulations of Systematic Reviews and Meta-Analyses. Hematology 2007; 493-7.
- Liberati A, Altman DG, Tetzlaff J, Mulrow C, Gøtzsche PC, Ioannidis JP, et al. The PRISMA statement for reporting systematic reviews and meta-analyses of studies that evaluate healthcare interventions: explanation and elaboration.BMJ. 2009; 339:b2700.
- Wells G, Shea B, O’connell J, Robertson J, Peterson J, Welch V, et al. The Newcastle-Ottawa Scale (NOS) for assessing the quality of nonrandomised studies in meta-analysis. 3rd Symposium on Systematic Reviews: Beyond the Basics, July 3–5; Oxford; 2000.
- Wong G, Greenhalgh T, Westhorp G, Pawson R. Realist methods in medical education research: what are they and what can they contribute? Medical Education. 2012; 46:89–96.
