# Supplementary material for: STORIES statement: Publication standards for healthcare education evidence synthesis
Source: BMC Med. 2014 Sep 3;12:143. doi: 10.1186/s12916-014-0143-0 (PMC4243720; doi:10.1186/s12916-014-0143-0)
Supplement: Additional file 2 — Comparison of STORIES statement with existing evidence synthesis checklists. [file 12916_2014_143_MOESM2_ESM.doc]

**Appendix 2:** Comparison with 4 other evidence synthesis statements with STORIES statement (novel or significant reworked items highlighted in grey)

| **PRISMA statement^25^** | **RAMESES^27^** | **Meta-analysis of individual participant data^28^** | **MOOSE group^29^** | **STORIES statement** |
| --- | --- | --- | --- | --- |
| **Title** | | | | |
| Identify the report as a systematic review, meta-analysis, or both. | In the title, identify the document as a realist synthesis or review |  | Identify the study as a meta-analysis (or systematic review) | Use a title that includes a description of the aims of the piece (educational effectiveness, descriptive, etc) and method of evidence synthesis (e.g. realist, meta-ethnographic, etc) |
| **Abstract** | | | | |
| Provide a structured summary including, as applicable: background; objectives; data sources; study eligibility criteria, participants, and interventions; study appraisal and synthesis methods; results; limitations; conclusions and implications of key findings; systematic review registration number | While acknowledging that requirements and house style may differ between journals, abstracts should ideally contain brief details of the study's background, review question or objectives; search strategy; methods of selection, appraisal, analysis and synthesis of sources; main results; and implications for practice. |  | Use the journal’s structured format | Provide a structured summary |
| Background / Introduction | | | | |
| Describe the rationale for the review in the context of what is already known | Explain why the review is needed and what it is likely to contribute to existing understanding of the topic area. |  | Present the clinical problem and hypothesis | Describe the rationale for the review in the context of what is already known |
| Provide an explicit statement of questions being addressed with reference to participants, interventions, comparisons, outcomes, and study design (PICOS). | State the objective(s) of the review and/or the review question(s). Define and provide a rationale for the focus of the review. |  | A statement of objectives that includes the study population, the condition of interest, the  exposure or intervention, and the outcome(s) considered | Provide a statement of the questions being addressed by the study |
|  | Explain why realist synthesis was considered the most appropriate method to use | Why the individual participant data approach was initiated |  | State why this method of evidence synthesis was selected within the context of the questions being asked |
| **Methods** | | | | |
| Indicate if a review protocol exists, if and where it can be accessed (e.g., Web address), and, if available, provide registration information including registration number. | Any changes made to the review that was initially planned should be briefly described and justified | Whether there was a protocol for the individual participant data project, and where it can be found |  |  |
| Specify study characteristics (e.g., PICOS, length of follow-up) and report characteristics (e.g., years considered, language, publication status) used as criteria for eligibility, giving rationale. | While considering specific requirements of the journal or other publication outlet, state and provide a rationale for how the iterative searching was done | The process used to identify relevant studies for the meta-analysis | Types of study designs considered | State and provide a rationale for how the searching was done |
| Describe all information sources (e.g., databases with dates of coverage, contact with study authors to identify additional studies) in the search and date last searched. | Provide details on all the sources accessed for information in the synthesis. For example, where electronic databases have been searched, details should include, for example, the name of the database, search terms, dates of coverage and date last searched. | How authors of relevant studies were approached for individual participant data | Search strategy, including time period included in the synthesis and keywords | Provide details on all the sources of information and dates searched |
| Present full electronic search strategy for at least one database, including any limits used, such that it could be repeated. |  | How many authors (or collaborating groups) were approached for individual participant data, and the proportion that provided such data | Databases and registries searched  · Search software used, name and version, including special features used (eg, explosion), Use of hand searching (eg, reference lists of obtained articles), List of citations located and those excluded, including justification | Electronic databases - provide full search terms for at least one database, with details of deviations in subsequent searches |
| State the process for selecting studies (i.e., screening, eligibility, included in systematic review, and, if applicable, included in the meta-analysis). | Explain how judgements were made about including and excluding data from documents, and justify these |  |  | Explain the method for judging inclusion / exclusion |
| Describe method of data extraction from reports (e.g., piloted forms, independently, in duplicate) and any processes for obtaining and confirming data from investigators. | Describe and explain which data or information were extracted from the included documents and justify this selection | How authors of relevant studies were approached for individual participant data, How many authors (or collaborating groups) were approached for individual participant data, and the proportion that provided such data, The number of authors who did not provide individual participant data, the reasons why, and the number of patients (and events) in the respective study | Method of addressing articles published in languages other than English, Method of handling abstracts and unpublished studies  Description of any contact with authors | Describe the process of data extraction and any process of contacting authors for confirmation of / or more data |
| List and define all variables for which data were sought (e.g., PICOS, funding sources) and any assumptions and simplifications made. |  | Documentation of how data were classified and coded (eg, multiple raters, blinding, and  interrater reliability), Assessment of confounding (eg, comparability of cases and controls in studies where  appropriate), Assessment of study quality, including blinding of quality assessors; stratification or  regression on possible predictors of study results |  |  |
| Describe methods used for assessing risk of bias of individual studies (including specification of whether this was done at the study or outcome level), and how this information is to be used in any data synthesis. |  |  | Assessment of study quality, including blinding of quality assessors; stratification or  regression on possible predictors of study results., Assessment of heterogeneity | If quality appraisal tools are used, please describe and justify their choice |
| State the principal summary measures (e.g., risk ratio, difference in means). |  |  |  |  |
| Describe the methods of handling data and combining results of studies, if done, including measures of consistency (e.g., I2) for each meta-analysis. | Describe the analysis and synthesis processes in detail. This section should include information on the constructs analyzed and describe the analytic process. | Whether there were any qualitative or quantitative differences between those studies providing individual participant data and those studies not providing individual participant data (if appropriate) | Statistical methods (eg, complete description of fixed or random effects models, justification  of whether the chosen models account for predictors of study results, dose-response models,  or cumulative meta-analysis) in sufficient detail to be replicated | Describe quantitative methods for synthesising primary evidence (where appropriate), such as meta-analysis and how issues of heterogeneity will be considered |
|  |  |  |  | Describe qualitative methods for synthesising primary evidence (where appropriate) and the goal of these methods, such as thematic analysis; meta-ethnography, and realist synthesis |
| Specify any assessment of risk of bias that may affect the cumulative evidence (e.g., publication bias, selective reporting within studies). |  |  |  |  |
| Describe methods of additional analyses (e.g., sensitivity or subgroup analyses, meta-regression), if done, indicating which were pre-specified. |  |  |  |  |
| **Results** | | | | |
| Give numbers of studies screened, assessed for eligibility, and included in the review, with reasons for exclusions at each stage, ideally with a flow diagram. | Provide details on the number of documents assessed for eligibility and included in the review with reasons for exclusion at each stage, as well as an indication of their source of origin (for example, from searching databases, reference lists and so on). |  | A table giving descriptive information for each included study | Give a flow diagram summarising study selection |
|  |  |  |  | If individuals familiar with the relevant literature and/or topic area were contacted, provide a summary of the contact and information obtained |
| For each study, present characteristics for which data were extracted (e.g., study size, PICOS, follow-up period) and provide the citations. | Provide information on the characteristics of the documents included in the synthesis. |  |  | Provide summarised details of included works, considering elements such as methodology, key results and conclusions |
| Present data on risk of bias of each study and, if available, any outcome level assessment (see item 12). |  |  | Assessment of quality of included studies | Describe methods of quality assessment of education reported, including all parameters considered (e.g. Details of study theoretical underpinning, pedagogical strategies and details of teaching activities to allow replication or dissemination) |
| For all outcomes considered (benefits or harms), present, for each study: (a) simple summary data for each intervention group (b) effect estimates and confidence intervals, ideally with a forest plot. |  |  |  |  |
| Present results of each meta-analysis done, including confidence intervals and measures of consistency. | Present the key findings with a specific focus on theory building and testing. | How many patients from each study were used in each meta-analysis performed, Whether the assumptions of the statistical models were validated (for example, proportional hazards) within each study, Whether the individual participant data results for each study were comparable with the published results, and, if not, why not (for example, individual participant data contained updated or modified information) | Indication of statistical uncertainty of findings | Present the results of qualitative and/or quantitative evidence synthesis |
| Present results of any assessment of risk of bias across studies (see Item 15). |  |  |  | Describe quality assessment of the research methods of included studies |
| Give results of additional analyses, if done (e.g., sensitivity or subgroup analyses, meta-regression [see Item 16]). |  |  | Results of sensitivity testing (eg, subgroup analysis) |  |
| Discussion | | | | |
| Summarize the main findings including the strength of evidence for each main outcome; consider their relevance to key groups (e.g., healthcare providers, users, and policy makers). | Summarize the main findings, taking into account the synthesis' objective(s), research question(s), focus and intended audience(s). Where applicable, compare and contrast the synthesis' findings with the existing literature (for example, other reviews) on the same topic |  | Consideration of alternative explanations for observed results | Present the main findings in light of the review objectives |
| Discuss limitations at study and outcome level (e.g., risk of bias), and at review-level (e.g., incomplete retrieval of identified research, reporting bias). | Discuss both the strengths of the review and its limitations. These should include (but need not be restricted to) (a) consideration of all the steps in the synthesis process and (b) comment on the overall strength of evidence supporting the explanatory insights that emerged |  | Strengths and weaknesses, Potential biases in the review process (eg, publication bias) | Discuss strengths and limitations of the review and its findings, commenting on the strength of the evidence |
| Provide a general interpretation of the results in the context of other evidence, and implications for future research. | List the main implications of the findings and place these in the context of other relevant literature. If appropriate, offer recommendations for policy and practice, The limitations identified may point to areas where further work is needed. |  | Generalization of the conclusions (ie, appropriate for the data presented and within the domain of the literature review), Guidelines for future research | Discuss how the findings of the evidence synthesis impact future primary research |
|  |  |  |  | Describe possible implications of the findings for educators |
| **Other** | | | | |
| Describe sources of funding for the systematic review and other support (e.g., supply of data); role of funders for the systematic review. | Provide details of funding source (if any) for the synthesis, the role played by the funder (if any) and any conflicts of interests of the reviewers | Whether ethics approval was necessary and (if appropriate) granted | Disclosure of funding source | Provide details of funding |
|  |  |  | Qualifications of searchers (eg, librarians and investigators) | Describe the skills and expertise of the review team and acknowledge any outside help |
